# Kidney disease progression and all-cause mortality across estimated glomerular filtration rate and albuminuria categories among patients with vs. without type 2 diabetes

**DOI:** 10.1186/s12882-020-01792-y

**Published:** 2020-05-07

**Authors:** Gregory A. Nichols, Anouk Derúaz-Luyet, Kimberly G. Brodovicz, Teresa M. Kimes, A. Gabriela Rosales, Sibylle J. Hauske

**Affiliations:** 1 Kaiser Permanente Center for Health Research, 3800 N. Interstate Avenue, Portland, Oregon, USA; 2 Boehringer Ingelheim International GmbH, Ingelheim am Rhein, Germany; 3 Boehringer Ingelheim Pharmaceuticals, Ridgefield, CT USA; 4 Vth Department of Medicine, University Medical Center Mannheim, University of Heidelberg, Heidelberg, Germany

**Keywords:** Epidemiology, Renal disease, Diabetes, Electronic health records

## Abstract

**Background:**

Studies of progression of kidney dysfunction typically focus on renal replacement therapy or percentage decline in estimated glomerular filtration rate (eGFR) as outcomes. Our aim was to compare real-world patients with and without T2D to estimate progression from and to clinically defined categories of kidney disease and all-cause mortality.

**Methods:**

This was an observational cohort study of 31,931 patients with and 33,201 age/sex matched patients without type 2 diabetes (T2D) who had a serum creatinine and urine albumin-to-creatinine ratio (UACR) or dipstick proteinuria (DP) values. We used the first available serum creatinine value between 2006 and 2012 to calculate baseline eGFR and categorized them and the corresponding UACR/DP values using the Kidney Disease Improving Global Outcomes (KDIGO) categories. To assess our primary outcomes, we extracted probabilities of eGFR progression or mortality from life-table analyses and conducted multivariable Cox regression analyses of relative risk adjusted for age, sex, race/ethnicity, smoking, ischemic heart disease, heart failure, and use of renal-angiotensin-aldosterone system inhibitors.

**Results:**

Patterns of eGFR decline were comparable among patients with vs. without T2D with larger percentage declines at higher albuminuria levels across all eGFR categories. eGFR decline was generally larger among T2D patients, particularly in those with severely increased albuminuria. Across all CKD categories, risk of progression to the next higher category of eGFR was substantially increased with increasing albuminuria. For example, the risk was 23.5, 36.2, and 65.1% among T2D patients with eGFR 30–59 ml/min/1.73m^2^ and UACR < 30, 30–299, and > 300 mg/dL, respectively (*p* < 0.001). Other comparisons were similarly significant. Among patients with low eGFR and normal to mildly increased albuminuria, the relative risk was up to 8-fold greater for all-cause mortality compared with the non-CKD subgroup (eGFR> 60 ml/min/1.73m^2^ with normal to mildly increased albuminuria).

**Conclusions:**

Presence of albuminuria was associated with accelerated eGFR decline independent of T2D. Risk for adverse outcomes was remarkably high among patients with CKD and normal to mildly increased albuminuria levels. Independent of T2D or albuminuria, a substantial risk for adverse outcomes exists for CKD patients in a routine care setting.

## Background

Chronic kidney disease (CKD) is a growing public health problem that is driven by an ageing population and the obesity epidemic [1]. The hallmark of CKD is low estimated glomerular filtration rate (eGFR) [2], but albuminuria also increases the risk of CKD and progression to end-stage kidney disease (ESKD), especially among patients with diabetes [3–5]. Even before progression to ESKD, CKD is associated with considerable morbidity [6], and patients with CKD are more likely to die than progress to ESKD [7]. Inhibition of the renin-angiotensin-aldosterone system (RAAS) with angiotensin-converting enzyme inhibitors (ACEi) or angiotensin receptor blockers (ARB) reduces albuminuria and slows the rate of progression in proteinuric kidney diseases, particularly in diabetes, and newer anti-hyperglycemic agents such as sodium-glucose cotransporter-2 (SGLT2) inhibitors and glucagon-like peptide-1 (GLP-1) receptor agonists have reno-protective effects [8, 9]. However, a substantial residual risk of ESKD remains [10–12].

Current estimates in the general population place US prevalence of CKD at approximately 15% [6]. Existing studies of CKD progression have typically examined only ESKD or mortality as outcomes [3, 13–16], or defined progression in terms of percentage decline in eGFR [17]. Although continuous measures of kidney function and kidney damage, eGFR and UACR values are typically categorized to aid clinical decision making [18, 19], but to our knowledge, no studies to date have compared rates of progression of kidney dysfunction from and to each of the clinically recognized categories in patients with and without type 2 diabetes (T2D) in a routine care setting. Moreover, despite being a risk factor for CKD progression [20], most studies either have not differentiated between patients with and without T2D [13, 15, 16] or stratified on diabetes status rather than directly comparing results [3]. One recent study compared trajectories of eGFR decline between people with and without T2D but did not address progression to poorer states of kidney function [21]. Comparing progression from and to clinical categories of kidney dysfunction and the incidence of ESKD and mortality in patients with and without T2D could have important implications for treatment, particularly among those without T2D and/or without albuminuria, about whom less is known. To fill these knowledge gaps, we conducted a comparative cohort study of real-world patients with and without T2D comparing progression from and to clinically defined categories of kidney disease and all-cause mortality.

## Methods

We conducted a longitudinal observational cohort study using the electronic health records (EHR) of Kaiser Permanente Northwest (KPNW), an integrated delivery system that serves approximately 550,000 individuals within a 75-mile radius of Portland, Oregon, USA. KPNW membership includes people whose insurance is self-paid, provided through employment, or by Medicare or Medicaid, and is representative of the service area [22].

For this study, we identified 39,295 patients with T2D who met the SUPREME-DM Study definition of diabetes prior to 2013 [23]. We matched them 1:1 to patients without T2D on age, sex, and year of the first serum creatinine value recorded in the EHR between January 2006 and December 2012. We excluded 5117 patients with and 4496 patients without T2D with no valid creatinine value in 2006–2012, and 2252 patients with and 1602 patients without T2D with no follow-up creatinine value, resulting in an analysis cohort of 31,931 patients with and 33,201 patients without T2D. At baseline, no patients were using SGLT2 inhibitors or GLP-1 receptor agonists.

### eGFR categories

We used the first available serum creatinine value between 2006 and 2012 recorded after diabetes recognition to calculate baseline eGFR using the CKD-EPI equation [24]; values < 60 mL/min/1.73m^2^ were confirmed by a second eGFR 3–12 months later. We categorized these values using the Kidney Disease Improving Global Outcomes (KDIGO) categories for eGFR (G1, normal or high: > 90 mL/min/1.73m^2^; G2, mildly decreased: 60–89 mL/min/1.73m^2^; G3a, mildly to moderately decreased: 45–59 mL/min/1.73m^2^; G3b, moderately to severely decreased: 30–44 mL/min/1.73m^2^; G4, severely decreased: 15–29 mL/min/1.73m^2^; and G5, kidney failure: < 15 mL/min/1.73m^2^ or diagnosis of ESKD defined as initiation of dialysis or kidney transplant). All renal dialysis is performed at contracted facilities and is captured in the KPNW claims database. For analysis purposes, we combined category G1 with G2, and category G3a with G3b.

### Albuminuria/proteinuria categories

KDIGO also recommends measurement of albuminuria via a urine albumin-to-creatinine ratio (UACR). However, in routine clinical practice UACR tests are not commonly performed among patients without T2D. To maximize sample size, we followed previous studies that combined UACR with dipstick proteinuria (DP) data to assess KDIGO categories of albuminuria [3, 15, 25, 26], creating the following categories: A1 (normal to mildly increased): UACR < 30 mg/g or DP negative or trace; A2 (moderately increased): UACR 30–299 mg/g or DP 1+; and A3 (severely increased): UACR > 300 mg/g or DP 2+ or higher. We required a UACR or DP to occur within 6 months of the baseline eGFR, and prioritized UACR over DP if both were available. We included patients with no baseline UACR/DP data available in the analysis of annualized continuous eGFR decline (where no UACR/DP was required) but excluded these patients from the analyses of progression of eGFR categories.

### Follow-up and outcomes

Following the baseline eGFR, we captured all eGFR measurements through December 2016. For each patient, follow-up for eGFR progression ended on the date of their last eGFR measurement but continued through 31 December 2016 to assess mortality. We calculated the adjusted annual rate of eGFR decline as the difference between baseline and last eGFR measurement divided by the time in days between those two measurements multiplied by 365 days, adjusted for age, sex, and number of observed eGFR measurements.

Among patients with baseline eGFR > 60 ml/min/1.73m^2^ (G1 or G2), we determined progression to moderate CKD (G3a or G3b), advanced CKD (G4) or ESKD (G5) based on the lowest eGFR recorded during follow-up that could be verified by a second eGFR value < 60 mL/min/1.73m^2^ within 90–365 days. Patients in whom progression could not be confirmed by a second value were determined to have remained in their baseline category. Similarly, patients with moderate CKD at baseline were evaluated for progression to advanced CKD or ESKD, and patients with advanced CKD were followed for progression to ESKD based on the lowest eGFR recorded during follow-up, dialysis, or transplant. All-cause mortality was assessed regardless of eGFR progression.

### Statistical methods

We used life table analysis to estimate eGFR transition probabilities with 95% confidence intervals and probabilities of all-cause mortality, reporting the probabilities at 1, 3 and 5 years of follow-up. This method replicates the analyses of nephropathy progression from the United Kingdom Prospective Diabetes Study [27] and describes the experience of the cohort as a whole unadjusted for risk factors.

We estimated the risk of progression to ESKD and all-cause mortality using Cox regression analysis adjusting for age, sex, non-white race/ethnicity, cigarette smoking, ischemic heart disease, heart failure, and use of a RAAS blockade agent. Ischemic heart disease and heart failure were defined by any encounter diagnosis prior to baseline using all available look-back. Use of RAAS blockade was defined as a dispense within 90 days prior to baseline with a days-supply that was beyond the baseline date. We used KDIGO-specified categories of low, moderate, high, and very high risk (see box), using non-T2D patients in the lowest risk category for reference [18]. All analyses were executed using SAS version 9.4 (SAS Institute, Cary, NC).

## Results

Due to matching, patients with and without T2D were of nearly identical age and sex (Table 1). Mean baseline eGFR was similar between the two cohorts, but the cohorts otherwise differed. Patients without T2D were less likely to have a UACR/DP measurement, while those with T2D were more likely to have severely increased albuminuria. T2D patients were significantly more likely to be Hispanic or non-Hispanic black, to smoke, and to have higher systolic blood pressure, higher BMI, lower LDL cholesterol, lower HDL cholesterol, and higher triglycerides. Patients with T2D were also significantly more likely to have existing comorbidities at baseline including ischemic heart disease, stroke, heart failure and depression. A much higher percentage of patients with T2D were receiving treatment with RAAS blockers and statins.

**Table 1:**
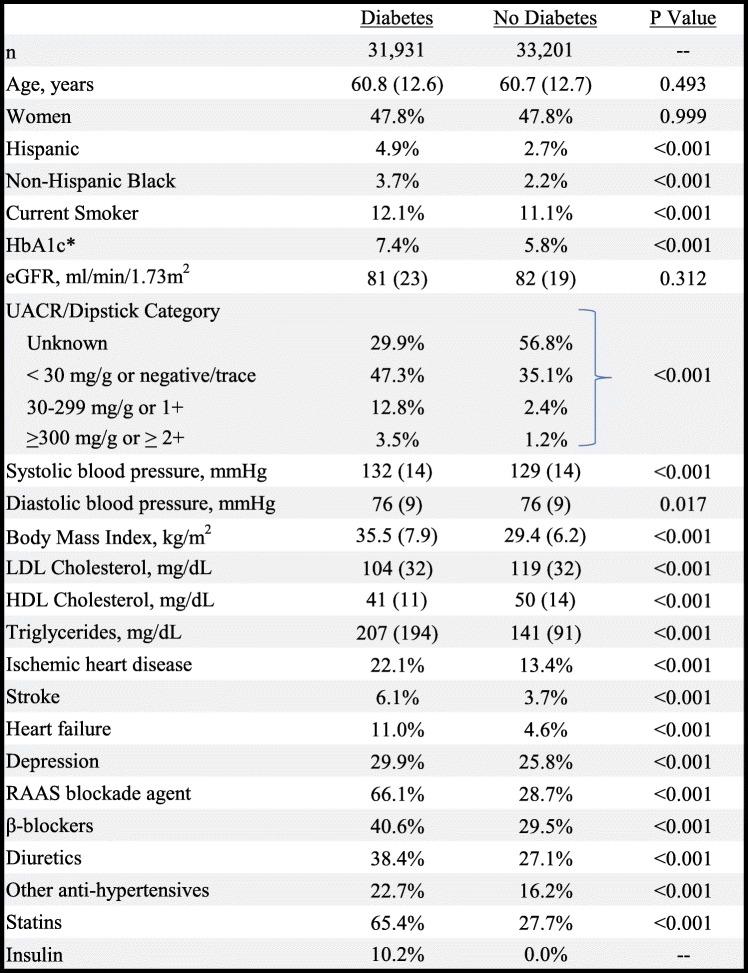
Baseline characteristics

*HbA1c data are missing for 86% of the patients without diabetes

Supplemental Table 1 compares age, sex, and use of RAAS blockers of patients with vs. without T2D by baseline eGFR and UACR/DP category; despite statistical significance, age and sex distributions were approximately similar. RAAS blocker use was generally higher among those with vs. without T2D.

### Percentage of eGFR decline

**Fig. 1:**
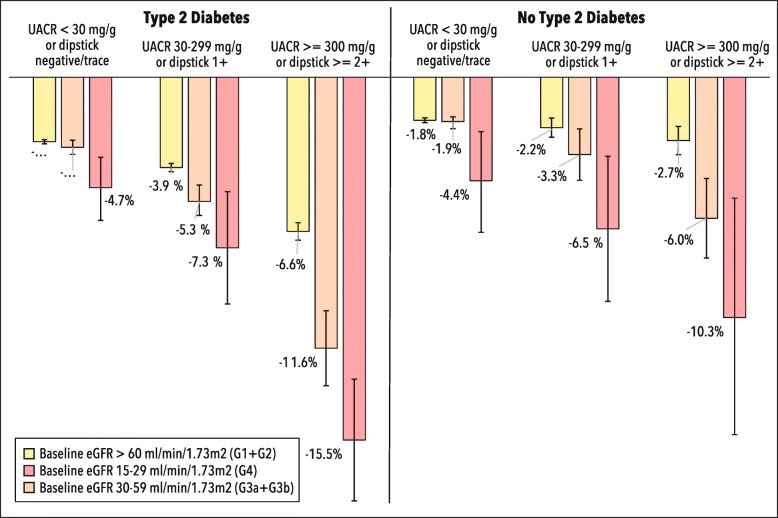
Adjusted annualized percentage decline in eGFR (with 95% error bars) by baseline eGFR and UACR/DP categories for patients with and without diabetes. Data are adjusted for age, sex, use of a RAAS blockade agent, and number of eGFR measurements

Independent of diabetes status, adjusted annualized percentage declines in eGFR increased with higher baseline albuminuria categories across all baseline eGFR categories (Fig. 1). eGFR declines were larger among patients with vs. without T2D. Adjusted annual percentage declines were greater at higher baseline eGFR categories (lower eGFR levels) regardless of T2D status. Crude annualized absolute and percentage declines showed the same pattern as the adjusted percentage declines (supplemental Table 2), and adjustment only modestly attenuated the values.

### eGFR progression and all-cause mortality

**Fig. 2:**
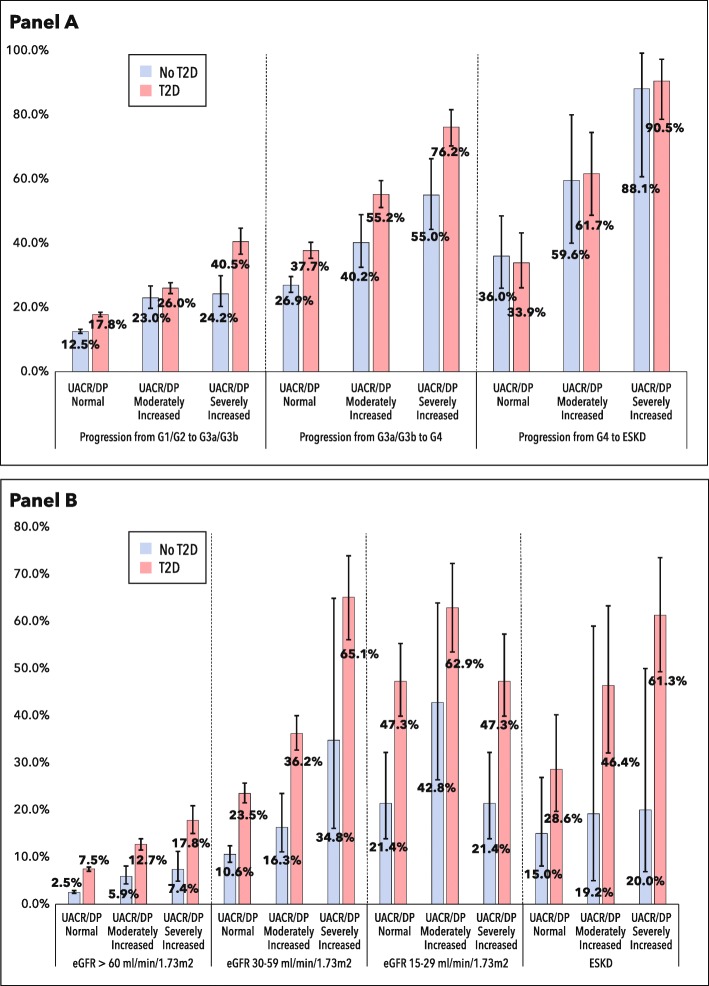
5-year probabilities of progression to the next higher category of eGFR (panel A) and of all-cause mortality (panel B). Probabilities and 95% error bars are extracted from life table analyses and are unadjusted

Figure 2 displays the crude probability of progression to the next more severe eGFR category (panel A) or all-cause mortality (panel B) over 5 years of follow-up. In all categories of eGFR and for all levels of albuminuria, T2D patients were 1.5 to 3 times more likely to die from any cause than patients without T2D. Among patients with eGFR> 60 or from 30 to 59 ml/min/1.73m^2^, those with T2D were more likely to progress to the next higher eGFR category compared with non-T2D patients. Progression from advanced CKD to ESKD was similar between T2D and non-T2D patients.

A subgroup of particular interest was patients with normal/mildly increased albuminuria but low eGFR levels (< 60 ml/min/1.73m^2^). Among all patients with CKD (low eGFR and/or moderately/severely increased albuminuria), this phenotype comprised 26% of those with and 52% of those without T2D. Overall, at comparable baseline eGFR categories, patients with moderately or severely increased albuminuria levels were at consistently higher risk of category progression or of all-cause mortality independent of diabetes status. However, regardless of T2D, for patients at low eGFR levels with normal to mildly increased albuminuria, the relative risk for progression to the next higher CKD category was 2–4-fold higher and up to 8-fold higher for mortality when compared to the respective non-CKD subgroup (eGFR> 60 ml/min/1.73m^2^ with normal to mildly increased albuminuria). One, three and five-year probabilities of progression and all-cause mortality, including probabilities of progressing more than one risk category, are displayed in supplemental Figs. 1, 2, and 3 respectively.

### Multivariable adjusted risk

**Fig. 3:**
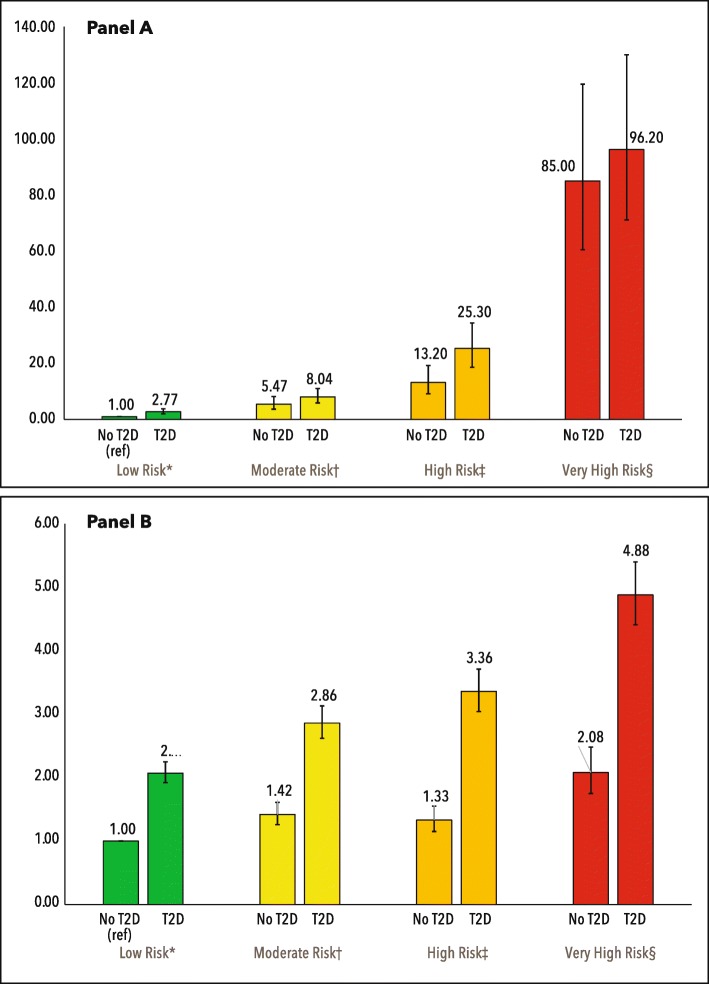
Hazard ratios (95% CI) for progression to ESKD (panel A) and all-cause mortality (panel B) by KDIGO risk categories among patients with vs. without type 2 diabetes. Green (reference) is low risk, yellow is moderate risk, orange is high risk, red is very high risk. Hazard ratios are adjusted for age, sex, non-white race/ethnicity, cigarette smoking, ischemic heart disease, heart failure, and use of a RAAS blockade agent

After statistical adjustment over median follow-up of 7.6 years (interquartile range 4.7–10.5), compared with low risk patients without T2D, those with T2D at moderate risk had an eight-fold increased risk of progression to ESKD (hazard ratio 8.04, 95% CI 5.90–10.96) while those with T2D at high risk had an increased relative risk of 25 times (Fig. 3a). Among patients without T2D and at moderate risk there was a more than five-fold increased risk of progression to ESKD (5.47, 3.67–8.16) and a 13-fold greater risk among high risk non-T2D patients. Patients with T2D in the low risk category had a nearly 3-fold increased risk of progression to ESKD compared with patients without T2D (2.77, 2.04–3.77).

Compared with patients without T2D at low risk, T2D patients at low risk were twice as likely to die (2.07, 1.92–2.25) while T2D patients at moderate, high or very high risk had hazard ratios (95% CI) for mortality of 2.86 (2.62–3.13), 3.36 (3.04–3.71), and 4.88 (4.41–5.40), respectively (Fig. 3b). Comparable numbers for patients without T2D were 1.42 (1.26–1.61), 1.33 (1.15–1.55), and 2.08 (1.75–2.48), respectively.

## Discussion

In this population-based cohort study of routine clinical care of 31,931 people with T2D and 33,201 people of similar age and sex without T2D, we found (unsurprisingly) that T2D and presence of albuminuria were strongly associated with kidney disease progression and mortality. Despite differences in risk rates, we found similarities in risk associated with albuminuria and the patterns of eGFR decline independent of T2D. We also found a high risk of adverse outcomes in patients with low eGFR and normal to mildly increased albuminuria.

T2D appeared to be a precipitating factor for incident CKD and for the deterioration of kidney function and for all-cause mortality. This is consistent with an Atherosclerosis Risk in Communities study showing an almost twice as rapid decline in eGFR among those with vs. without diabetes [21]. Our data indicate this relationship holds true across all categories of baseline eGFR as well as categories of albuminuria/proteinuria despite much greater use of RAAS blockers among T2D patients. The accelerated progression of eGFR associated with T2D was observed at all stages of baseline eGFR in both adjusted and unadjusted analyses. Given the greater mortality risk observed among patients with T2D at earlier eGFR stages, their risk of progression may be understated. Thus, as life expectancy of people with diabetes lengthens, the incidence of CKD and ESKD can be expected to increase.

The independent role of albuminuria/proteinuria in predicting risk of progression to ESKD and mortality is well-recognized [18, 28]. Our data underscore the powerful impact of albuminuria/proteinuria on these outcomes, regardless of T2D. The Chronic Kidney Disease Prognosis Consortium (CKDPC) conducted 5 meta-analyses of progression to ESKD or mortality comparing people in general [15, 26], with and without diabetes [3], with and without hypertension [16], and among patients with prevalent chronic kidney disease [29]. The results of these meta-analyses were reported as adjusted rates or hazard ratios with different categorizations, making direct comparisons to our results difficult. Consistent with our results, each of the CKDPC meta-analyses found progressively increased risk of CKD progression [15], ESKD [3, 15, 29], and mortality [3, 26, 29] at each successive baseline category of eGFR as well as each successive albuminuria/proteinuria category. Also consistent with our results, patients with diabetes were more likely to progress to ESKD [3]. In addition, the rates of eGFR decline we report are similar to a recent report from the Steno Diabetes Center and consistent with their findings that albuminuria status affects eGFR decline with the steepest decline among persons with macroalbuminuria [30].

Despite greater risk associated with T2D, the *patterns* of progression and mortality were similar for patients with and without T2D across baseline categories of eGFR and UACR/DP but the difference between patients with and without T2D in progression rates to each subsequently higher category grew in a stepped fashion. In addition to T2D, several other risk factors associated with the incidence and progression of CKD have been identified, including age, sex, glycemic control, blood pressure, non-white race, obesity, smoking, HDL cholesterol, cardiovascular disease and depression [31]. In our data, all of these risk factors were significantly more prevalent among T2D patients. These risk factors are sometimes but not always statistically significant predictors of progression of kidney dysfunction, yet their inclusion in multivariable models do not typically improve model discrimination beyond that achieved with eGFR alone [31, 32]. The covariates we tested were significant predictors of progression to ESKD or all-cause mortality, but did not substantially impact the hazards we report. Therefore, the relative differences between T2D and non-T2D patients cannot be explained by risk factors other than diabetes, although residual confounding may remain. Furthermore, our adjusted analyses demonstrate a substantially increased risk of CKD progression and all-cause mortality associated with baseline eGFR and albuminuria among non-T2D patients.

Accumulating data indicate a shift in the clinical course of CKD towards a phenotype with normal to mildly increased albuminuria, mainly evident in T2D patients [26]; low eGFR in the absence of increased urine albumin excretion has become more prevalent [33, 34]. Such patients have a relatively low risk for kidney disease progression and ESKD but a clear association with cardiovascular disease and mortality risk [35, 36]. Our data underscore the existing evidence that the risk for kidney disease progression and mortality is substantially higher in patients with severely increased albuminuria levels than in patients with normal to mildly increased levels across all eGFR categories. However, among patients with normal to mildly increased albuminuria the relative risk for progression to the next higher eGFR category was 2–4-fold higher and all-cause mortality up to 8-fold higher for patients with low vs. normal eGFR, independent of T2D. Our findings add to the growing evidence of a transformation of the clinical course of CKD towards a non-proteinuric/albuminuric phenotype in which the increased risk for adverse outcomes maybe underappreciated.

Our study has limitations. Because UACR measurements were rarely performed in patients without T2D, we included proteinuria ascertained from urine dipstick tests to define our albuminuria categories. The distribution of use of UACR and dipstick measures differed between those with and without T2D. We cannot determine how this affected our results but a similar approach has been used in other studies [3, 15, 25, 26]. We calculated change in eGFR from only two measurements. Because eGFR levels are known to have substantial inherent variability, our method may result in some inaccurate estimates, a problem that would be somewhat mitigated by the large sample size. The adjusted annual percentage declines we report assume a linear decline and may not account for the inherent variability in eGFR measurements. However, our method of estimating eGFR decline is clinically meaningful and similar to the primary outcome used in the REPRISE study (Replicating Evidence of Preserved Renal Function: An Investigation of Tolvaptan Safety and Efficacy) [37, 38]. Categorization of UACR/DP could result in patients with different continuous values of UACR being included in the same albuminuria category. Extracting probabilities from life table analysis does not allow for covariate adjustment and therefore represent the experience of the current cohort only. However, our large sample size from a real-world clinical setting is typical of a U.S. clinical population in general [39], and what differences do exist are unlikely to affect the relative contributions of baseline eGFR and albuminuria to disease progression or mortality. Moreover, the adjusted Cox regression analyses of ESKD progression and all-cause mortality *increased* the unadjusted relative difference between patients with and without T2D, suggesting our unadjusted estimates are conservative. Nevertheless, potential unmeasured confounding is a limitation of observational studies, although inclusion of additional available confounders such as blood pressure and anti-hypertensive use other than RAAS inhibitors did not appreciably alter our findings. Current guidelines recommend inclusion of a 25% decline in eGFR to determine progression [19]. We did not include percentage decline in eGFR when determining progression because this would have created follow-up eGFR categories that those same guidelines do not recognize [18]. We determined eGFR categories from creatinine values collected at irregular intervals in the course of routine clinical care. We confirmed low eGFR values with a second measurement, but low values could not always be confirmed, in which case the patient remained in the previously observed (less severe) category. The misclassification would generate conservative estimates of categories of kidney dysfunction. All patients were required to have a baseline and at least one additional follow-up creatinine value. Though common for T2D patients, creatinine tests for people without T2D may have been ordered during a clinical workup of a medical condition. If so, our non-T2D sample may have been less healthy than the general population, thus overestimating their baseline prevalence of CKD and progression rates. However, this would result in an underestimate of the rate ratios comparing people with and without T2D. Although we did not account for use of SGLT-2 inhibitors or GLP-1 receptor agonists during follow-up, use of these agents is extremely low in our study setting (< 5%) and cannot account for the differences we observed.

## Conclusions

In summary, the pattern of decline of kidney function was similar for a population-based cohort of patients with and without T2D in a routine care setting. T2D was associated with an increased prevalence and incidence of CKD, and an accelerated and increased risk of kidney disease progression despite much greater use of RAAS blockers. For all KDIGO risk categories, the mortality risk was about two-fold higher in patients with T2D vs. without T2D, including patients in the lowest risk category. The subgroup of patients with CKD and normal or mildly increased albuminuria showed a remarkably increased risk for kidney disease progression or all-cause mortality compared to the respective group with no CKD independent of diabetes status. Though the risks for adverse outcomes were significantly smaller compared with patients with moderately or severely increased albuminuria, the risk should not be neglected in clinical practice. To date, there are no specific treatment options beyond optimization of risk factors for the management of CKD patients with normal or mildly increased albuminuria, accentuating the high unmet medical need for new treatment options. In secondary analyses of cardiovascular outcome trials, SGLT2 inhibitors and GLP-1 receptor agonists have demonstrated renoprotection among people with type 2 diabetes [8, 9]. However, most clinical trials examining interventions to slow kidney disease progression are limited to individuals with T2D and/or those with CKD and severely increased albuminuria levels, yet our findings suggest that most patients with CKD have normal to mildly increased albuminuria levels. More evidence from clinical trials among patients without as well as with diabetes is urgently needed to advance treatment across the broad range of CKD phenotypes.

## Supplementary information

**Additional file 1: Table S1.** Comparison of age, sex, and use of RAAS blockade for all patients with and without diabetes in each baseline eGFR and UACR/DP category. **Table S2.** Crude annualized absolute and percent decline in eGFR by baseline eGFR and UACR/DP categories among patients with a baseline and follow-up eGFR with and without diabetes. **Figure S1.** 1-year probabilities (95% CI) of progressing to a higher eGFR stage and of all-cause mortality by baseline eGFR and UACR/DP categories for patients with and without diabetes. The probabilities are based on life table analysis over a maximum follow-up of 11 years. **Figure S2.** 3-year probabilities (95% CI) of progressing to a higher eGFR stage and of all-cause mortality by baseline eGFR and UACR/DP categories for patients with and without diabetes. The probabilities are based on life table analysis over a maximum follow-up of 11 years. **Figure S3.** 5-year probabilities (95% CI) of progressing to a higher eGFR stage and of all-cause mortality by baseline eGFR and UACR/DP categories for patients with and without diabetes. The probabilities are based on life table analysis over a maximum follow-up of 11 years.

## Data Availability

The datasets analyzed during the current study were derived from the proprietary data systems of KPNW and are not publicly available but are available from the author subject to organizational stipulations.

## Abbreviations

ACEi: Angiotensin converting enzyme inhibitot
ARB: Angiotensin receptor blockers
BMI: Body mass index
CKD: Chronic kidney disease
CKD-EPI: Chronic kidney disease epidemiology collaboration
CKDPC: Chronic kidney disease prognosis consortium
DP: Dipstick proteinuria
eGFR: Estimated glomerular filtration rate
EHR: Electronic health records
ESKD: End-stage kidney disease
GLP-1: Glucagon-like peptide-1 receptor agonists
HDL: High-density lipoprotein
KDIGO: Kidney disease improving global outcomes
KPNW: Kaiser Permanente Northwest
LDL: Low-density lipoprotein
RAAS: Renin-angiotensin-aldosterone system
REPRISE: Replicating Evidence of Preserved Renal Function: An Investigation of Tolvaptan Safety and Efficacy
SGLT2: Sodium-glucose co-transporter 2 inhibitors
SUPREME-DM: Surveillance Preventjon and Management of Diabetes Mellitus
T2D: Type 2 diabeters
UACR: Urine albumin to creatinine ratio
