# Correction to: Kidney disease progression and all-cause mortality across estimated glomerular filtration rate and albuminuria categories among patients with vs. without type 2 diabetes

**DOI:** 10.1186/s12882-020-01854-1

**Published:** 2020-05-28

**Authors:** Gregory A. Nichols, Anouk Déruaz-Luyet, Kimberly G. Brodovicz, Teresa M. Kimes, A. Gabriela Rosales, Sibylle J. Hauske

**Affiliations:** 1 Kaiser Permanente Center for Health Research, 3800 N. Interstate Avenue, Portland, Oregon, USA; 2 Boehringer Ingelheim International GmbH, Ingelheim am Rhein, Germany; 3 Boehringer Ingelheim Pharmaceuticals, Ridgefield, CT USA; 4 Vth Department of Medicine, University Medical Center Mannheim, University of Heidelberg, Heidelberg, Germany

**Correction to: BMC Nephrology (2020) 21:167.**

**https://doi.org/10.1186/s12882-020-01792-y**

Following publication of the original article [1], we were notified that one of the coauthors has her name mis-spelled.

Incorrect: Anouk Derúaz-Luyet

Correct: Anouk Déruaz-Luyet

The original article has been corrected.
