# Supplementary material for: Kidney disease progression and all-cause mortality across estimated glomerular filtration rate and albuminuria categories among patients with vs. without type 2 diabetes
Source: BMC Nephrol. 2020 May 7;21:167. doi: 10.1186/s12882-020-01792-y (PMC7203828; doi:10.1186/s12882-020-01792-y)
Supplement: Supplementary file 1 — Additional file 1: Table S1. Comparison of age, sex, and use of RAAS blockade for all patients with and without diabetes in each baseline eGFR and UACR/DP category. Table S2. Crude annualized absolute and percent decline in eGFR by baseline eGFR and UACR/DP categories among patients with a baseline and follow-up eGFR with and without diabetes. Figure S1. 1-year probabilities (95% CI) of progressing to a higher eGFR stage and of all-cause mortality by baseline eGFR and UACR/DP categories for patients with and without diabetes. The probabilities are based on life table analysis over a maximum follow-up of 11 years. Figure S2. 3-year probabilities (95% CI) of progressing to a higher eGFR stage and of all-cause mortality by baseline eGFR and UACR/DP categories for patients with and without diabetes. The probabilities are based on life table analysis over a maximum follow-up of 11 years. Figure S3. 5-year probabilities (95% CI) of progressing to a higher eGFR stage and of all-cause mortality by baseline eGFR and UACR/DP categories for patients with and without diabetes. The probabilities are based on life table analysis over a maximum follow-up of 11 years. [file 12882_2020_1792_MOESM1_ESM.docx]

**Supplemental Material**

Table S1. Comparison of age, sex, and use of RAAS blockade for all patients with and without diabetes in each baseline eGFR and UACR/DP category.

Table S2. Crude annualized absolute and percent decline in eGFR by baseline eGFR and UACR/DP categories among patients with a baseline and follow-up eGFR with and without diabetes.

Figure S1. 1-year probabilities (95% CI) of progressing to a higher eGFR stage and of all-cause mortality by baseline eGFR and UACR/DP categories for patients with and without diabetes. The probabilities are based on life table analysis over a maximum follow-up of 11 years.

Figure S2. 3-year probabilities (95% CI) of progressing to a higher eGFR stage and of all-cause mortality by baseline eGFR and UACR/DP categories for patients with and without diabetes. The probabilities are based on life table analysis over a maximum follow-up of 11 years

Figure S3. 5-year probabilities (95% CI) of progressing to a higher eGFR stage and of all-cause mortality by baseline eGFR and UACR/DP categories for patients with and without diabetes. The probabilities are based on life table analysis over a maximum follow-up of 11 years.
